# Nanoparticles-mediated CRISPR-Cas9 gene therapy in inherited retinal diseases: applications, challenges, and emerging opportunities

**DOI:** 10.1186/s12951-022-01717-x

**Published:** 2022-12-03

**Authors:** Yueh Chien, Yu-Jer Hsiao, Shih-Jie Chou, Ting-Yi Lin, Aliaksandr A. Yarmishyn, Wei-Yi Lai, Meng-Shiue Lee, Yi-Ying Lin, Tzu-Wei Lin, De-Kuang Hwang, Tai-Chi Lin, Shih-Hwa Chiou, Shih-Jen Chen, Yi-Ping Yang

**Affiliations:** 1 Department of Medical Research, Taipei Veterans General Hospital, 201, Section 2, Shi-Pai Road, Taipei, 11267 Taiwan, ROC; 2 Institute of Pharmacology, National Yang-Ming Chiao Tung University, Taipei, 11221 Taiwan; 3 School of Medicine, National Yang-Ming Chiao Tung University, Taipei, 11221 Taiwan; 4 Department of Medicine, Kaohsiung Medical University, Kaohsiung, 80708 Taiwan; 5 Doctoral Degree Program of Translational Medicine, National Yang Ming Chiao Tung University and Academia Sinica, Taipei, 11221 Taiwan; 6 Department of Ophthalmology, Taipei Veterans General Hospital, Taipei, 11217 Taiwan; 7 Department of Internal Medicine, Taipei Veterans General Hospital, Taipei, 11217 Taiwan; 8 Institute of Food Safety and Health Risk Assessment, National Yang-Ming University, Taipei, 11221 Taiwan; 9 Genomic Research Center, Academia Sinica, Taipei, 11529 Taiwan

**Keywords:** CRISPR-Cas9, Inherited retinal disease, Nanoparticle, Retinal organoid, Patient-derived iPSCs

## Abstract

Inherited Retinal Diseases (IRDs) are considered one of the leading causes of blindness worldwide. However, the majority of them still lack a safe and effective treatment due to their complexity and genetic heterogeneity. Recently, gene therapy is gaining importance as an efficient strategy to address IRDs which were previously considered incurable. The development of the clustered regularly-interspaced short palindromic repeats (CRISPR)-CRISPR-associated protein 9 (Cas9) system has strongly empowered the field of gene therapy. However, successful gene modifications rely on the efficient delivery of CRISPR-Cas9 components into the complex three-dimensional (3D) architecture of the human retinal tissue. Intriguing findings in the field of nanoparticles (NPs) meet all the criteria required for CRISPR-Cas9 delivery and have made a great contribution toward its therapeutic applications. In addition, exploiting induced pluripotent stem cell (iPSC) technology and in vitro 3D retinal organoids paved the way for prospective clinical trials of the CRISPR-Cas9 system in treating IRDs. This review highlights important advances in NP-based gene therapy, the CRISPR-Cas9 system, and iPSC-derived retinal organoids with a focus on IRDs. Collectively, these studies establish a multidisciplinary approach by integrating nanomedicine and stem cell technologies and demonstrate the utility of retina organoids in developing effective therapies for IRDs.

## Introduction

Inherited retinal diseases (IRDs) are a diverse group of rare genetic disorders associated with more than 280 different genes [1]. IRDs manifest varying degrees of clinical severity and variable inheritance patterns [2], leading to blindness in infancy/early childhood [3, 4] or a gradual and progressive vision loss during adulthood [5–8]. The development of comprehensive and effective treatment proves to be a challenge for scientists, particularly due to the diverse number of genes involved in IRDs. In 2017, Luxturna^®^ (voretigene neparvovec), a gene therapy drug developed by Spark Therapeutics Inc., was approved by the Food and Drug Administration (FDA). Luxturna^®^ uses adeno-associated virus serotype 2 (AAV2) as a delivery vehicle to carry the wild-type *Retinal Pigment Epithelium 65* (*RPE65*) gene into the retinal cells with *RPE65* mutation for treating patients with Leber congenital amaurosis (LCA), a rare form of inherited vision loss [9]. AAV-derived vectors have several advantages, including high biosafety, low immunogenicity, stable expression, and high infectivity in several cell types. Although AAV-derived vectors are the safest and most effective viral vectors for gene replacement therapy in the retina, they cannot accommodate genes larger than 4.7 kb, and the generation of neutralizing antibodies against AAV may attenuate the efficacy of AAV-mediated gene therapy [10, 11]. Moreover, the treatment with Luxturna^®^ requires vitrectomy of the retina, followed by the retinal detachment using the air tamponade [12, 13]. This multi-step surgical procedure is a huge burden to patients’ fragile retinas. Another concern is the repeated treatments with Luxturna^®^, as a single Luxturna^®^ dosage only lasts for five years [14] and patients are required to undergo these invasive procedures routinely. Therefore, innovative topical delivery and highly permeable gene therapy are urgently needed for the therapy of IRDs [15].

The development of the clustered regularly-interspaced short palindromic repeats (CRISPR)-CRISPR-associated protein 9 (Cas9) gene editing technique revolutionized molecular biology and showed great potential for improved gene therapy [16, 17]. The first clinical trial of CRISPR-Cas9 gene editing treatment for IRDs, delivered by the AAV, was launched for the most common cause of inherited childhood blindness, LCA type 10 (LCA10) [18]. Homology-independent targeted integration (HITI) is an advanced CRISPR-Cas9 technique that enables targeted gene insertion in non-dividing cells and presents a new approach to treating genetic disorders [19]. However, one of the greatest challenges in the therapeutic application of CRISPR-Cas9 for retinal diseases is the delivery efficiency of the CRISPR-Cas9 components into the retinal pigment epithelium (RPE) and the neurosensory retinal environment in the posterior pole of human eyeballs [19]. The gene delivery using viral vectors is efficient but associated with several disadvantages, including random insertion, mutagenesis, and biohazard concerns [20]. Recent developments in nanomedicine overcome this difficulty by introducing a nontoxic delivery of CRISPR-Cas9 components that can significantly alleviate safety concerns raised by viral vectors [20]. Today, researchers aim to engineer nanoparticles (NPs) with specialized properties to go beyond viral limitations and create new opportunities towards the application of CRISPR-Cas9 in treating IRDs. However, the translation of such technology to the clinic is hampered by several obstacles. The complexity of the retinal structure poses a significant challenge to the standard measurement of visual performance after treatment and causes unreliable diagnosis [21, 22]. Moreover, in vitro, in vivo*,* and species variations among disease models limit the ability to fully recapitulate the structure and functions of the retina [23–25]. With induced pluripotent stem cells (iPSC) technology's burgeoning field, researchers are now generating three-dimensional (3D) retinal organoids (ROs) from human iPSCs [26–31], aiming at recapitulating disease phenotypes and developing a clinically relevant resource to investigate the molecular mechanisms of diseases. This review highlights current nanomedicine applications in gene therapy, focusing on an NP-mediated delivery of the CRISPR-Cas9 system with respect to IRDs. Meanwhile, we elaborate on the application of iPSC-derived retinal organoids as a drug-screening platform, which, in combination, provide new opportunities for drug development and personalized medicine for IRDs.

## The complex three-dimensional (3D) architecture of the retina

**Fig. 1:**
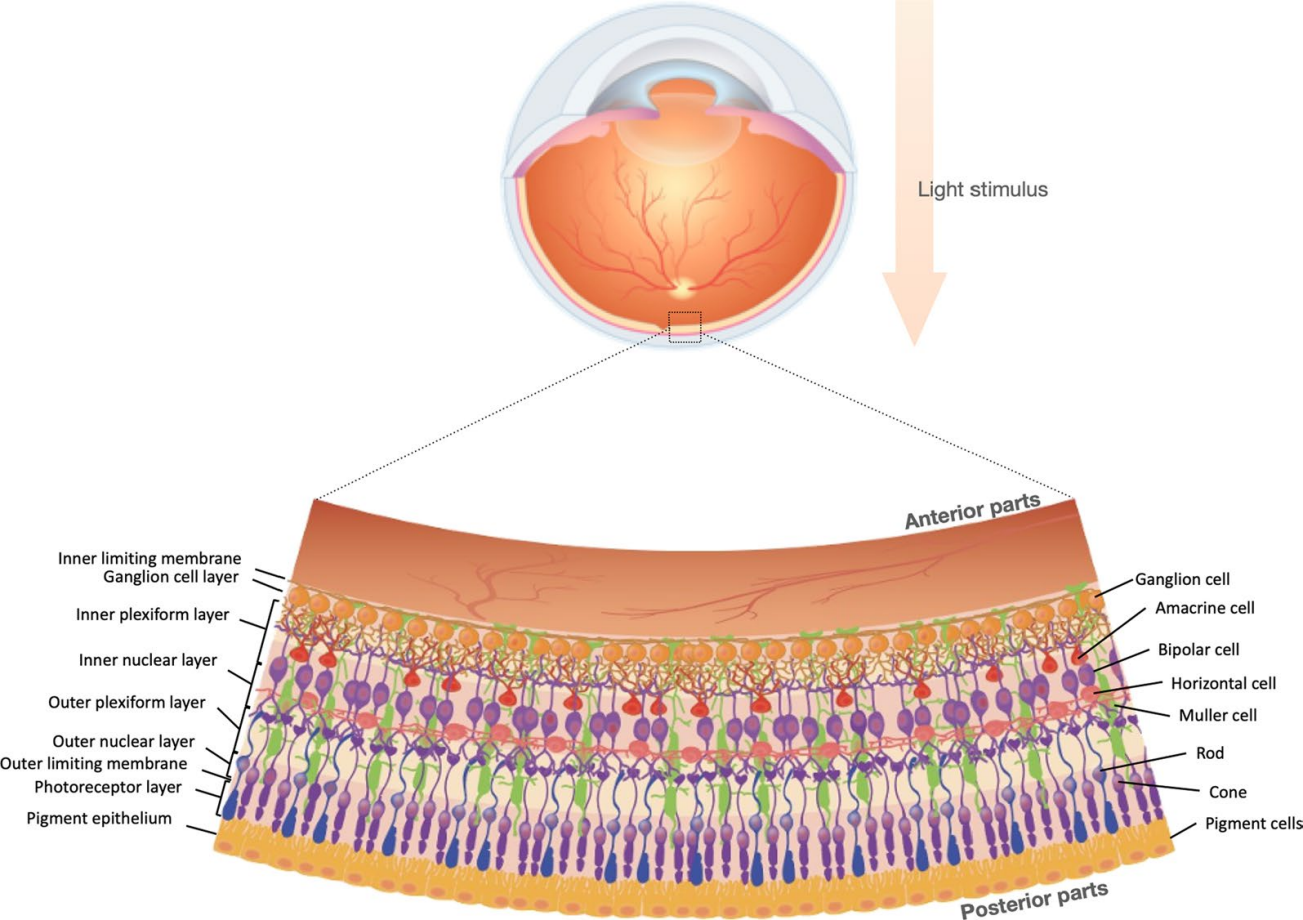
The complex architecture of the retina. The general layout of the retinal layers is shown on the left and cell types on the right. Photoreceptor cells, bipolar cells, and retinal ganglion cells constitute the signal transmission pathway that conveys vision signal to the brain. Horizontal and amacrine cells are interneurons modulating visual signal transmission. Müller glial cells perform the neuronal support functions similar to those of astrocytes in the brain

During embryonic development, the retina is derived from the prosencephalon, the anterior portion of the brain [21]. The retina’s unique architecture can be classified into two distinct parts: the posterior RPE layer with the most apparent light absorption function and the anterior multilayered neuroretina (Fig. 1). This multi-layered structure of the retina comprises two synaptic layers (the outer and inner plexiform layers) and three specialized neuronal cell layers (Fig. 1). The first neuronal layer comprises rod and cone photoreceptor (PR) cells that convert the absorbed light with different intensities into electrical signals via phototransduction. PR cells then form synapses with the second layers of neuronal cells, horizontal and bipolar cells present in the inner nuclear layer (INL), through the outer plexiform layer (OPL). This eventually leads to the transmission of the signal from PRs to the third class of neuronal layer cells, the retinal ganglion cells (RGCs), through the inner plexiform layer (IPL) (Fig. 1). Finally, the axons of RGCs converge to form the optic nerve, which in turn leads to the transmission of the visual impulses from the eye to the brain [21, 22]. Any impairment in this signaling cascade can result in visual disorder. In addition, the photoreceptor dysfunction or loss can be associated with age, diabetes, and genetics [32–34]. The latter causes a specific category of disease described as IRDs, which is the focus of this review.

## Inherited retinal diseases and the treatment obstacles

IRDs associated with several clinically and genetically heterogeneous defects belong to a group of progressive retinal degeneration diseases that may lead to vision loss. They exhibit a wide variation in genetic mutations, age of onset, and disease severity [35–37], with an estimated incidence of 1 in 2000 to 3000 individuals [38–42]. Due to recent advances toward pathogenesis and characterization of genes responsible for IRDs, with more than 270 genes identified so far, a significant progress has been made in the field of incurable IRDs [43–48]. It has led to the development of treatments aimed at restoring vision or delaying the vision loss progression; however thus far, treatment options are still limited. IRD gene variants can be transmitted in an autosomal dominant (e.g. LCA and retinitis pigmentosa (RP)) [49, 50], recessive (e.g. cone-rod dystrophy) [51], and X-linked manner (e.g. X-linked juvenile retinoschisis (XLRS)) [52–55]. As shown in Table 1, IRDs can be clinically classified into six categories based on the affected retinal regions/cell types and four categories depending on the genetic inheritance mode; other classifications are based on the monogenic and multifactorial nature of IRD and the disease progression [56]. The genetic heterogeneity observed in these diseases manifests in patients with very similar clinical phenotypes but different genetic diagnoses, demanding gene-specific therapies or gene editing treatments to develop treatment strategies [8, 57]. Nevertheless, there is no available cure for IRDs currently, and ophthalmology has been at the forefront of utilizing gene therapy to treat these disorders.

**Table 1:** Classification of IRDs

IRD	Autosomal dominant	Autosomal recessive	X-linked	Mitochondrial inheritance
Diffuse photoreceptor dystrophies				
Cone-rod dystrophy	✓	✓	✓	
Retinitis pigmentosa	✓	✓	✓	✓
Leber congenital amaurosis	✓	✓		
Enhanced S cone disease		✓		
Congenital stationary night blindness	✓	✓	✓	
Achromatopsia		✓	✓	
Choroidal dystrophies				
Choroideremia			✓	
Gyrate atrophy		✓		
Bietti crystalline dystrophy		✓		
Macular dystrophies				
Best vitelliform dystrophy	✓	✓		
Stargardt disease		✓		
Sorsby macular dystrophy	✓			
Inner retinal dystrophies				
Retinoschisis			✓	
Hyaloid retinopathies				
Wagner disease	✓			
Knobloch syndrome		✓		
Familial exudative vitreoretinopathy	✓			
Associated with systemic symptoms				
Bardet-Biedl syndrome		✓		
Usher syndrome		✓		
Stickler syndrome	✓	✓		

IRDs can be classified by various genetic inheritance modes and affected retinal regions/cell types

## Overview of gene therapy techniques for IRDs

Gene therapy application for ophthalmic diseases is a blooming field of research and currently overcoming the barriers for translation to the clinic, which is extensively described in multiple reviews [58–63]. AAV vector-based gene therapy has obtained the marketing approval for treating *RPE65-*associated LCA [18, 64–66], Leber hereditary optic neuropathy (LHON) [67, 68], and choroideremia (CHM) [69–71]. Treatments for LHON and CHM have entered phase III clinical trials and raised hopes that these approaches might be of practical use to delay or halt disease progression in patients with these IRDs [72–74]. Currently, more than 30 gene therapy trials for IRDs are being conducted in the United States, and some have entered the phase III clinical trials. Positive outcomes in these clinical trials were mainly due to the advantages offered by the retina as the target organ and AAV as the carrier [75–78]. The eye is an ideal target for gene therapy approaches due to several features of its anatomy and microenvironment. First, the eye is an immune-privileged microenvironment in which the tight junctions of the blood-retina barrier (BRB) limit systemic dissemination of intraocularly injected antigens, making it suitable for the introduction of viral vectors. Besides, BRB plays a crucial role in regulating the retina’s microenvironment as its enclosed orbit environment allows a small number of vectors encoding the gene editing components to reach the optimal therapeutic concentration with the desired effect. Secondly, the accessibility of the eye enables the intravitreal and subretinal delivery of vectors and allows the non-invasive monitoring of the patient's response to therapy [79–81]. Lastly, an indefinite expression of delivered genes in non-dividing retinal cells can potentially be achieved only after a single injection, which is a tremendous advantage to non-integrating vectors [82].

As previously mentioned, gene therapy can be conducted using viral vectors to introduce the transgene into the target cells. The most broadly used vectors for ocular gene delivery are AAVs [83]. AAVs are favored for gene therapy as they are non-pathogenic helper-dependent viruses with little immunogenicity and high diffusion capacity. They are also capable of efficient transduction of quiescent retinal cells and allow the long-term expression of the transgene in the host cells. However, their restricted packaging size of approximately 4.7 kb limits their application [84, 85]. Despite many reports on the higher efficiency of viral versus non-viral vectors [86–88], recent findings on NP-based delivery for retinal gene therapy have proven to be more efficient than their viral counterparts [89, 90]. Current DNA-based retinal therapy strategies involve gene augmentation/replacement and CRISPR-Cas9-mediated gene editing, as discussed below.

### Gene augmentation/replacement therapy

Current gene therapy strategies targeting IRDs rely on gene augmentation/supplementation in which the wild copy of the gene is delivered to affected cells resulting in a functional protein product. In most retinal gene therapies, the transgene is introduced using the AAV vector and delivered via a subretinal injection after the vitrectomy [56, 91, 92]. In 2017, the FDA approved the first gene therapy using Luxturna^®^ for treating a monogenic disease—biallelic *RPE65-*related retinal dystrophy. Luxturna^®^ treatment delivers a functional copy of the *RPE65* gene to the retina of patients suffering from severe night blindness (nyctalopia). It can significantly improve patient’s light sensitivity and the navigation in dim light. Among all IRDs, slowly progressing IRDs are more favorable for gene therapy as it can only target viable cells. In patients with advanced disease when most of the photoreceptors are degenerated, photoreceptor replacement therapy appears to be more attractive approach to restore vision [61, 73, 74, 93]. Furthermore, disorders with monogenic X-linked or autosomal recessive mutations are usually the potential targets for gene therapy, as such therapy is ineffective for disorders with gain-of-function mutations in which the mutant protein products can interfere with the functions of wild type proteins [94, 95]. Therefore, advanced technologies are required to overcome the limitations associated with the gene augmentation and broaden the scope of retinal gene therapy.

### CRISPR-Cas9 genome engineering

The efficient genome editing relies on the formation of DNA double-strand breaks (DSBs) at the desired genomic loci by an engineered nuclease and the subsequent repair of DSBs, exploiting two main mechanisms: the homology-directed repair (HDR), a precise gene editing strategy using DNA template with homology arms [96], and the non-homologous end-joining (NHEJ), an error-prone process which may lead to insertions or deletions (indels) at the break site. Nuclease-based platforms including zinc finger nucleases (ZFNs) and transcription activator-like effector nucleases (TALENs) have been extensively used for generating precise DSBs in cells over the past few decades. However, the technical shortcomings and laborious protein engineering impose certain limitations on their in vivo applicability.

In 2012, the advent of CRISPR-Cas9 technology with multiplexed gene editing ability and high efficiency opened up new horizons to a novel type treatment, genome surgery [97, 98]. The programmable RNA-guided Cas9 endonuclease targets and cleaves DNA of any size to insert or remove DNA fragments in a sequence-dependent manner without affecting the gene's regulatory sequences. Unlike gene augmentation, CRISPR-Cas9-mediated gene editing avoids the risk of toxicity caused by the transgene overexpression and overcomes the AAV packaging limits by using smaller Cas9 variants (e.g., *Staphylococcus aureus* Cas9 (SaCas9) [99] and *Streptococcus pyogenes* Cas9 [100]), making in vivo genome surgery an achievable goal. Furthermore, this strategy enables eliminating genes with dominant gain-of-function mutations, not amenable to gene augmentation therapy. Thus, CRISPR-Cas9 provides ophthalmologists with a novel therapeutic tool for treating IRDs unreachable by established treatments [101–104]. The CRISPR-Cas9 system can function via either the NHEJ or HDR pathways. The latter is preferable but inefficient and not readily accessible to post-mitotic cells. In contrast, Cas-induced NHEJ strategy is efficient and active in both dividing and non-dividing cells. Therefore, increasing CRISPR-Cas9 efficiency is a critical task to make CRISPR a broadly applicable gene therapy approach for treating IRDs.

Recently, several advanced strategies, such as obligate ligation-gated recombination (ObLiGaRe) [105, 106], homology-independent targeted integration (HITI) [107–109], precise integration into target chromosome (PITCH) [105, 110], base editing [111], prime editing [112], and CRISPR activation/interference [113], have been developed aiming at genome editing. However, the in vivo application of these strategies should be further validated before their clinical translation. HITI strategy was demonstrated to be able to bypass the low efficiency of HDR to a significant extent. Despite the development of CRISPR-Cas9, the targeted integration of transgenes in vivo remains mostly challenging, especially in most adult tissues that are non-dividing. HITI, designed by Suzuki et al., is known as the gene knock-in strategy in which the foreign DNA is directly ligated to DSBs, which can be achieved in both dividing and non-dividing cells via the NHEJ pathway [114–116]. Generally, following the HITI strategy, the CRISPR-Cas9-mediated gene knockin can be carried out as follows. First, Cas9 sgRNA is used to specifically recognize and process the targeted sequences in both genomic and donor DNA. Second, after generating the formation of three double-stranded breaks, DNA repair initiates the site-specific integration of transgenes via the NHEJ pathway. For instance, Suzuki’s study demonstrated the promising utility of HITI strategy that can promote efficient CRISPR-Cas9-mediated gene knock-in in the brain and eye in vivo [108]. The genomic safe harbors are the sites which can be safely manipulated, allowing the integrated transgene to function properly without affecting the genome of host cells [117]. Further studies showed that HITI strategy can be used to safely and efficiently integrate transgenes into zebrafish and mammalian cells [114–116].

Comparing to HDR and NHEJ, microhomology-mediated end-joining (MMEJ) that requires relatively much smaller regions for DSB repair is an alternative form of end-joining. Sakuma and Yamamoto et al. created an alternative method for gene knock-in, termed PITCH. Assisted by MMEJ, the PITCH system is able to promote precise gene knock-in with the requirements of much smaller homologous regions. Therefore, complicated cloning of homology arms was not needed, facilitating the entire process of PITCH vector construction [110]. Base editing, developed by Komor and Liu et al., is able to achieve programmable genome editing with direct and irreversible conversion of a specific DNA base into anthor through a mechanism that does not require the cleavage of dsDNA backbone or the donor template [111]. Base editors that can convert the target DNA base within an ~ 5-nucleotide window is capable of gene correction in several point mutations in human genetic diseases [111]. Prime editing was designed to achieve efficient gene correction with the minimal formation of byproducts. This technology uses a prime editing guide RNA (pegRNA) and a fusion protein consisting of a catalytically impaired Cas9 endonuclease and an engineered reverse transcriptase. Through the coordination of pegRNA and the fusion protein, prime editing can recognize the target sites and undergo the desired gene correction. Comparing to HDR, prime editing carries the minimal production of byproducts but shows similar or higher efficiency. Without the requirements of DSBs and the donor DNA templates, prime editing can mediate targeted insertion, deletion, and transversions which may largely expand the capabilities of gene editing [112]. Nuclease-deactivated Cas9 (dCas9) is designed for RNA-guided genomic transcription regulation. This technology can be employed in both CRISPR-Cas9 interference (CRISPRi) and CRISPR-Cas9 activation (CRISPRa) to achieve transcription regulation. In CRISPRi, once the dCas9 binds to the DNA target without cleaving it, either transcription initiation or elongation will be blocked, leading to sequence-specific repression of gene expression. In CRISPRa, the dCas9 interacts or is fused with transcription activators, eventually leading to the upregulation of specific genes [118]. Together, CRISPRi and CRISPRa can provide precision control of gene expression, instead of relying on genome editing.

**Fig. 2:**
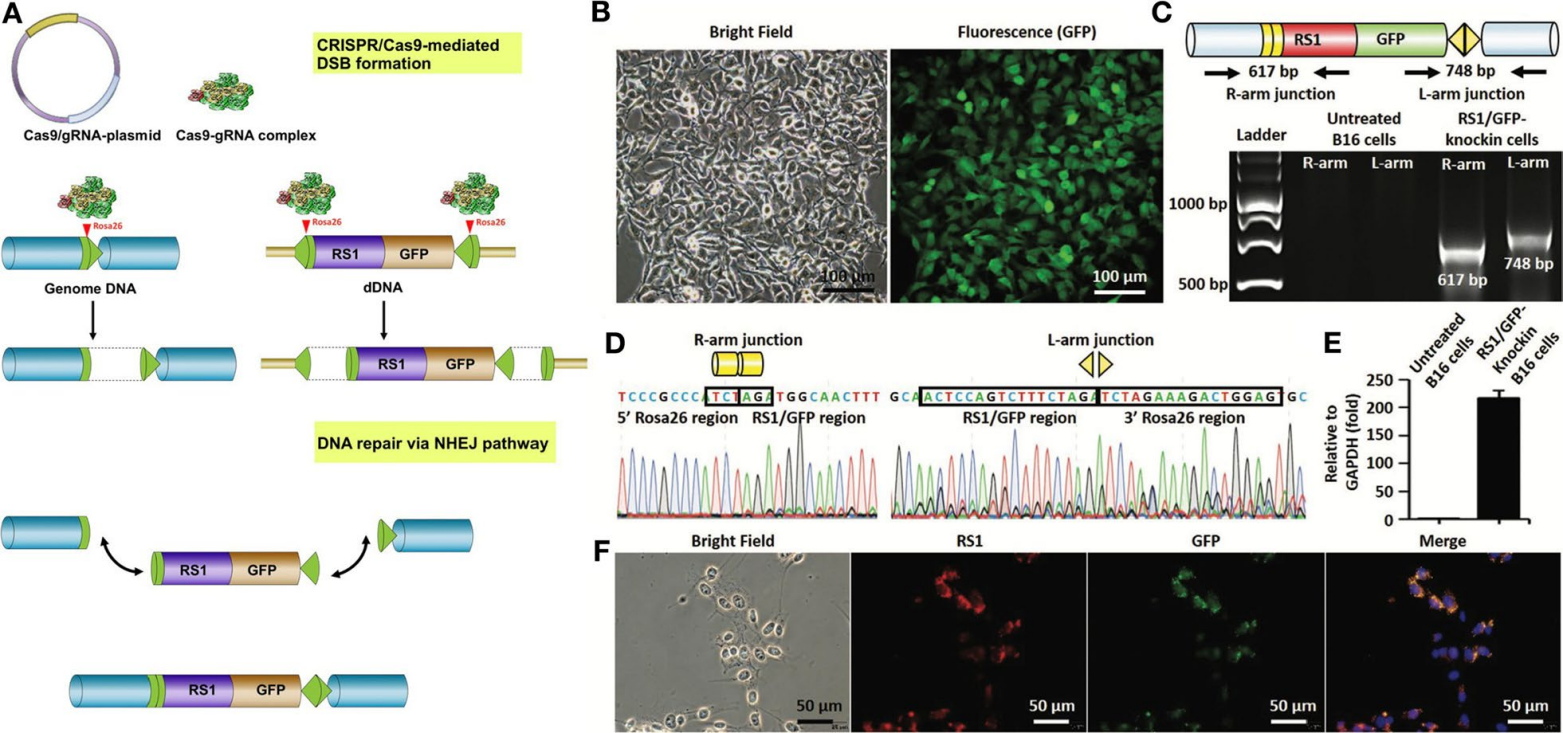
CRISPR-Cas9-mediated gene knock-in using the homology-independent targeted integration (HITI) strategy. **A** The CRISPR-Cas9-mediated gene editing initiates after the delivery of CRISPR-Cas9 machinery into target cells. The HITI strategy consists of two steps, including CRISPR-Cas9-mediated DSB formation and DNA repair via the NHEJ pathway. **B**–**D** An example of RS1/GFP knock-in using the HITI strategy. **B** Representative bright-field and fluorescence images of RS1/GFP-knock-in B16 cells. **C** PCR analysis showing the presence of right-arm (R-arm) junction (617 bp) and left-arm (L-arm) junction (748 bp) after the integration of RS1/GFP into the ROSA26 sites. **D** Sanger sequencing of the genome-donor boundaries in the R-arm and L-arm junctions confirming the integration of RS1/GFP. **E** Quantitative PCR analysis showing the upregulation of RS1 gene after the RS1/GFP gene knock-in. **F** Immunofluorescence staining showing the expression of RS1 and GFP after the RS1/GFP gene knock-in. All data are reproduced from our previous work [119]

Considering the potential of CRISPR-Cas9 system as a promising tool for precise genome manipulation, several studies have focused on its application in different IRD models. For example, we previously reported that HITI strategy could integrate the *RS1* gene into the mouse retina, providing a potential therapeutic solution for treating X-linked juvenile retinoschisis (XLRS; Fig. 2) [119], an IRD with a typical retinoschisis phenotype [82, 120, 121]. Bakondi et al. used CRISPR-Cas9 system to ablate a mutation in *Rho* gene which causes progressive photoreceptor loss and restored the normal gene function [122]. Other two studies further showed that the Cas9-induced NHEJ strategy can potentially prevent the progression of dominant monogenic diseases such as rhodopsin-associated retinitis pigmentosa (RP) and Best disease [122, 123]. More recently, CRISPR-mediated HDR approach has been adopted to validate the preservation of visual functions by correcting *Pde6b* mutations in mouse photoreceptors [124]. Yang et al. used CRISPR-generated mutant keratinocytes to identify the essential role of *EXOSC2* mutation in the pathogenesis of short stature, hearing loss, retinitis pigmentosa and distinctive facies (SHRF) syndrome [125]. To examine the utility of CRISPR-Cas9 gene editing on retinal dystrophies in vivo, the first phase I/II trial (NCT03872479) was conducted in March 2019 [126]. LCA type 10 is an IRD with IVS26 point mutation that creates a de novo splicing donor site and leads to a functional loss in the CEP290 protein [12]. The efficacy of the CRISPR medicine AGN-151587 (EDIT-101) to remove the IVS26 point mutation was evaluated and this trial was scheduled to be completed in 2024 [126]. EDIT-101 uses two gRNAs, taking advantage of NHEJ, creating a deletion to eliminate the IVS26 mutation [126]. For the aforementioned gene editing strategies, base editing has been used in effectively correct the C625T mutation and disease phenotypes in patient-derived retinal organoids [30] and to restore RPE65 expression and visual functions in a mouse model of IRD [127]. The in vivo efficiency of prime editing was found to be much lower than its efficiency [128, 129]. The large size of prime editing machinery also limited its in vivo delivery to the target. Liu et al. used dual adeno associated virus-mediated delivery of intein prime editing machinery to achieve in vivo delivery and gene editing in the mouse liver [129]. However, the dual vector systems and low efficiency remains concerns regarding the use of prime editing in vivo. It was reported that CRISPRi showed promising efficacy in the treatment of autosomal dominant IRDs [130], whereas the variable efficacy of CRISPRi-mediated gene knockdown, poor delivery efficiency in post-mitotic cells, and the unknown immune responses that may be caused by dCas9 may be the potential challenges for the clinical application of CRISPRi [130].

While the CRISPR technology has a great potential for improving current treatments for IRDs, an efficient and safe delivery system is a critical prerequisite for its success. In the next section, we discuss the significance of nanomaterials for the therapeutic application of CRISPR systems for potential gene therapy of IRDs.

## Nanoparticles (NPs)

**Fig. 3:**
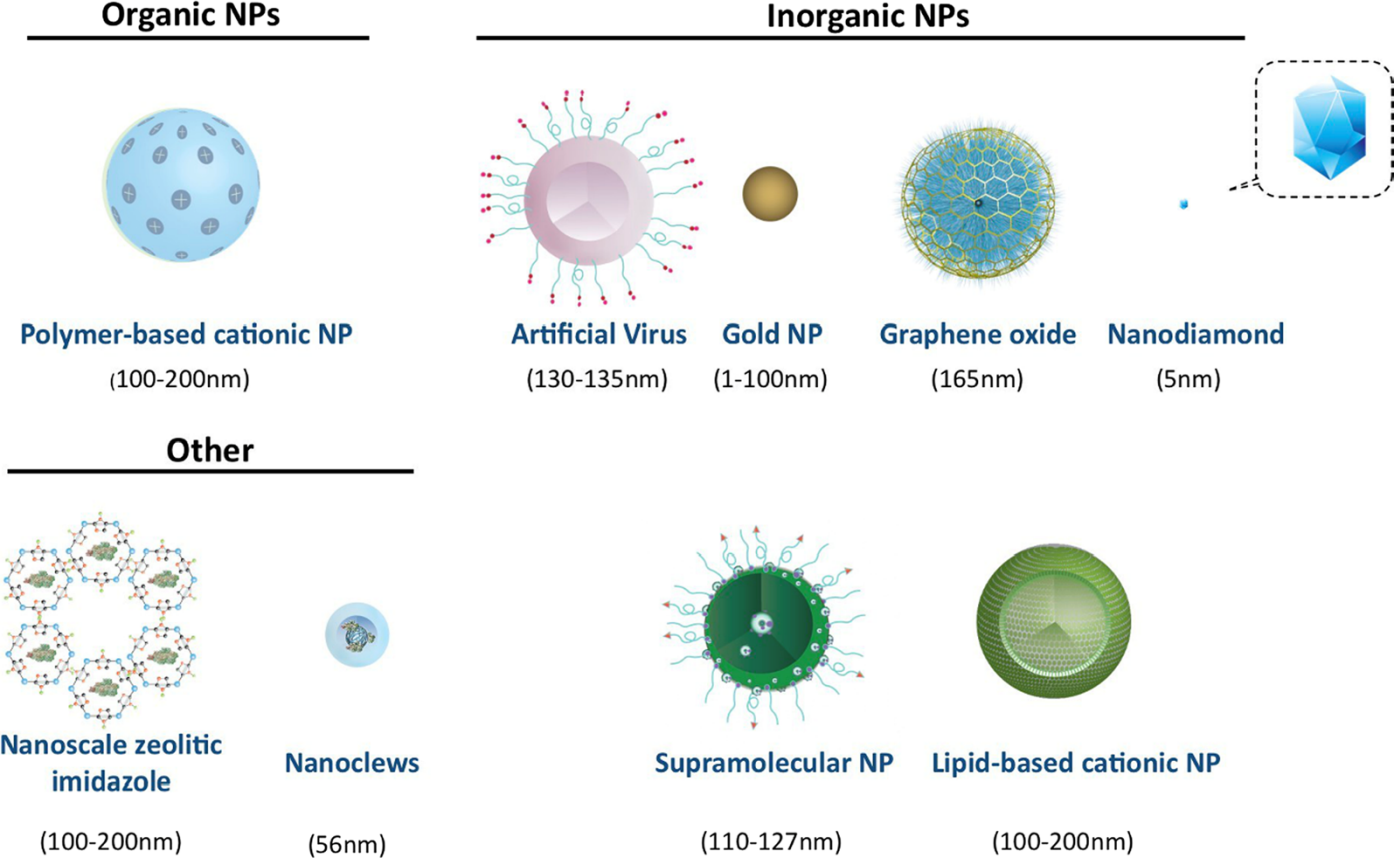
Classifications of NPs. NPs are classified into organic, inorganic, and other NPs. The average sizes of the particles are shown relative to each other and the structural features are shown as discussed in the article

Successful gene editing requires the efficient delivery of CRISPR-Cas9 machinery to the desired cells. To date, AAV is the most widely adopted delivery system targeting the retina and the eye. Despite Luxturna^®^ as the FDA-approved recombinant AAV gene therapy product [131], there are still some challenges in gene therapy using AAV-based vectors to efficiently deliver the transgenes. As mentioned earlier, the limited virus packaging size of AAV (4.7 kb) can hardly accommodate a typical Cas9 from *Staphylococcus Pyrogens* (approximately 4.2 kb in size) popularly used in CRISPR-Cas9 studies [10, 11]. In addition, the application of conventional AAV2 has been reported to raise immunogenicity concerns [132]. Several efforts and modifications in vector engineering have been made to improve the AAV-based gene delivery, including the improvement of AAV transduction efficiency, the tropism of AAV vectors, the reduction of the immunogenicity of AAV capsid and transgene, and the optimization of large-scale AAV manufacture [133]. To overcome the size limit of AAV and deliver a large gene expression cassette, scientists have attempted to split transgenes and deliver them via two or three individual AAV vectors [134]. However, the lower transduction efficiency using split AAV vectors than that of a single AAV vector is still a matter of concern [135]. Also, it costs 425,000 US dollars per eye to receive Luxturna^®^ treatment, imposing a heavy economic burden on the society or individuals [1]. Another concern in treating IRDs is the structural peculiarity of the retina, which demands a specific administration route depending on the choice of drug vehicles. With the advent of nanoparticle (NP)-mediated delivery, the unmet needs of efficient genome editing associated with viral vectors are expected to be greatly fulfilled. In therapeutics, the application of NPs as delivery carriers for genes and drugs has been profoundly investigated [136–141]. The classification of NPs based on different sizes and structures is shown in Fig. 3. Their nanoscale size enables them to interact with biological systems at the molecular level. In addition, numerous reports have documented that NPs can ensure successful targeted delivery and be transported across biological barriers that can make them an indispensable tool for the drug delivery [142–145]. The NP-mediated delivery of high molecular weight CRISPR-Cas9 complexes is one of the most significant approaches being developed for genome editing and other evolving applications [146–149]. Here, we review the promising gene delivery carriers with the potential for IRD treatment based on properties like the nanocarriers and load capacity.

To effectively deliver a therapeutic agent to the retina, the particle size and charge are important parameters when developing nanocarriers [150]. The inner limiting membrane (ILM) with a negative charge located between the vitreous and the retina is a physical and electrostatic barrier [151], which limits the diffusion of NPs [151, 152]. The diffusion of NPs or drugs into the retina varies due to the architecture differences among species, including mice, bovines, and humans [23–25]. For example, the pore size of human ILM is about 10 nm, with the variable thickness ranging from 100 nm in the fovea to 4 μm in the thickest area [23]. However, the thickness of the ILM in small animals (such as mice and rats) is less than 100 nm, so the pharmacokinetic distribution of drugs observed in animals often cannot be applicable in clinics [24]. In addition to ILM, the vitreous and retinal cell membranes are all negatively charged. Therefore, after the injection of NPs into the vitreous, the NP’s charge generates an electrostatic interaction which affects the diffusion rate of NPs within the vitreous. Furthermore, the charge of NPs also affects the permeability across the ILM. Therefore, excessive positive and negative charges are not conductive for the drug delivery to the retina [153]. Huang et al. compared lipid NPs with different charges (− 30mv ~  + 50mv) and found that + 35mv lipid NPs can achieve the highest distribution efficiency in the retina [154]. Here, we mainly focus on recent applications of NPs for delivering CRISPR-Cas9 to the retina while an extensive discussion of NP synthesis and design is beyond the scope of this article and has already been reviewed by others [155–165].

### Organic NP

#### Polymer-based cationic NPs

One of the well-known crucial properties of cationic polymers is to form the particular polymer/DNA polyplex. Cationic polymers carry no hydrophobic moiety, making them completely soluble in an aqueous solution. They can also compress DNA molecules to a small size which is considered a crucial feature for improving transfection efficiency in gene transfer [166]. These cationic polymer-based NPs coat negatively charged nucleic acids via electrostatic interactions that secure the nucleic acid's structural stability [158, 167]. Considerable progress has been made over the last decade in polymer-mediated CRISPR-Cas9 plasmid delivery for genome editing applications [149, 168–171]. The most widely used cationic polymers for pharmaceutical applications include poly-L-lysine (PLL), polyethylenimine (PEI), polyamidoamine (PAMAM) dendrimers, polyethylene glycol (PEG), and chitosan. Despite some evidences revealing the toxicity of PLL[172], PEG [173], PEI [174], and PAMAM [175], remarkable efforts have been made to modify and optimize these cationic polymers and render them as ideal vectors for effective gene delivery with lower toxicity, especially for the delivery of CRISPR-Cas9 [171, 176–178]. To date, no study reported the delivery of CRISPR-Cas9 machinery to the retina using this cationic polymer-based delivery strategy. In the following sections, we discuss the update on the main polymer-based NPs that have shown potential in the in vivo and in vitro delivery of CRISPR-Cas9. The application of chitosan in gene delivery is significantly limited due to its low transfection efficiency [179]. Thus, we exclude it from the discussion here.

The cationic polymer PAMAM is a spherical dendrimer composed of repeating branched subunits of amide and amine functional groups. PAMAM is classified into different “generations” (G) based on the number of surface groups that determine its size [180]. The higher the generation number, the greater the number of branches and surface positive charges. This enables PAMAM to interact with nucleic acids by electrostatic interactions and form polyplex complexes [180]. These branched architectures are extensively used in supramolecular chemistry as will be briefly discussed later [149, 181–187]. PAMAM dendrimers exhibit "proton sponge effect" that causes endosomal swelling and DNA release into the cytoplasm [146]. In several studies, PAMAM dendrimer has been shown to serve as an effective delivery system. Due to its unique characteristics, it can enhance the loading capacity, protect the drugs from degradation, and lessen systematic toxicity [188]. Yavuz et al. conjugated drugs with PAMAM to increase the drug release and ocular residence time and reported that PAMAM was safe for the retina and could be metabolized within 3 h after administration [189, 190]. Kretzmann et al. constructed a highly controllable dendronized polymer that consists of PAMAM dendrons and a linear copolymer backbone to deliver small and large plasmid DNA. This dendronized polymer is also capable of transfecting genome editing tools such as zinc fingers, transcription activator-like effectors (TALEs), and CRISPR/dCas9 platforms [191]. As for the CRISPR/dCas9 platform, Kretzmann et al. used this dendronized polymer to deliver CRISPRa that contains dCa9 fused to VP64 transactivator domain to achieve transcriptional activation of MASPIN (mammary serine protease inhibitor) in the MCF-7 breast cancer cells [191]. These findings highlighted the high transfection efficiency and packing capacity of the dendronized polymer that can deliver larger constructs. However, further in vivo studies are required to confirm its application. PAMAM dendrimers have also been used in CRISPR-Cas9 gene editing in HCT-116 and HT-29 cells [192] and to promote a CRISPR-Cas9-mediated gene editing of programmed death protein-1 (PD-L1) to obtain tumor immunotherapy in melanoma B16F10 cells [193]. So far there was no published studies using PAMAM dendrimers to deliver CRISPR-Cas9 machinery into the retina.

PEG is a polymer of choice for drug delivery applications, and it is often used to modify different nanoparticles [194–197]. PEG modification that involves the covalent conjugation of PEG to NPs, also called PEGylation, has been shown to enhance structural stability, electrostatic binding, and hydrophobicity. The conjugated protein can be tuned to specifically meet the requirements of drug delivery, for example, by increasing the solubility and stability of the drug while also reducing immunogenicity. This prolongs the retention time of the drug and the conjugate in the blood, thereby reducing the frequency of administration. Charged side chain polypeptide-based NPs [e.g., poly(lactic-co-glycolic acid) (PLG), PLL] have been investigated as drug and gene delivery vehicles [198–206]. However, their applications are limited due to the low water solubility and processing complications of these structures.

Previous studies demonstrated the efficient delivery of nucleic acids (such as siRNA) into cells using α-helical polypeptides [198–207]. A cationic α-helical poly-glutamate-based polypeptide, poly(γ-4-((2-(piperidin-1-yl)ethyl)aminomethyl)-benzyl-l-glutamate (PPABLG) was demonstrated to condense siRNAs or plasmids and maintain the helical structure that ensured high membrane penetration capacity for the cell entrance and endosomal escape. Furthermore, PPABLG was shown to protect the helical structure against environmental stress such as proteases, denaturing conditions, and pH [208]. In 2018, Wang et al. modified PPABLG by incorporating PEG-polythymine 40 (PEG-T 40) to generate PEGylayted helical polypeptide NPs (P-HNPs), 100 nm NPs, for the delivery of the CRISPR-Cas9 gene editing components. These NPs carried Cas9 and gRNA expression plasmids and delivered these components into various cell type to achieve efficient gene editing in vitro. They also used this P-HNP delivery system to achieve a CRISPR-Cas9-mediated gene disruption and repression of tumor growth in vivo [208]. The intratumoral injection of P-HNPs loaded with Cas9 DNA and sgRNA into the xenograft-transplanted mice model could target the survival gene, *Plk*. Although the reduction of tumor growth required ten injections, multiple administrations did not cause any weight loss in mice, indicating that P-HNPs lacked cytotoxicity. In-depth sequencing analysis of the tumor confirmed that the final genome editing efficiency was 35%, suggesting that P-HNPs may have the potential for medical applications [141].

PEI is one of the most notable cationic polymers with a large amount of positive surface charge exploited in gene transfection in vitro and in vivo [209]. PEI-modified NPs bind with and condense DNA to form spherical structures, which were fused with the endosome and elicited the “proton sponge effect” to escape endosomal degradation [210]. Several studies have emphasized on the application of this polymer in gene therapy [211–213]. In 2015, PEI was utilized for delivering Cas9 and gRNA for the first time, leading to the knockout of the *Ptch1* gene in the cerebellum of newborn mice [214]. Liang et al. encapsulated the CRISPR-Cas9 plasmid into an aptamer-functionalized PEG-PEI-Cholesterol nanocarrier to target and reduce vascular endothelial growth factor A (*VEGF*) gene expression both in vivo and in vitro [168]. However, the moderate toxicity of PEG-PEI-Cholesterol in subsequent clinical trials limited its application for drug delivery [215].

Liao et al. reported that the intravitreal injection of PEI/DNA polyplexes could deliver plasmids into retinal ganglion cells in mice. However, the efficiency of PEI-mediated gene editing was low [216]. β-cyclodextrin (β-CD), an FDA-approved drug [217], is a cyclic oligosaccharide with a diameter of 200 nm and high transfection efficiency for small plasmids [171, 218]. To increase the efficiency of gene transfection by PEI, this polymer was covalently linked with β-CD [219, 220] and showed no reported cytotoxicity in HEK293 cells [221]. This strategy increased the transfection efficiency of the luciferase gene to nearly four folds, compared with PEI alone [171]. Given that β-cyclodextrin-PEI (PEI/β-CD) can condense large plasmids at a high nitrogen-to-phosphorus ratio (N/P ratio; the ratio of positively-chargeable polymer amine (N) groups to negatively-charged nucleic acid phosphate (P) groups), Zhang et al. evaluated the efficiency of PEI/β-CD-mediated delivery of CRISPR-Cas9 system in HeLa cells in which the gene transfection efficiency was about 34%. Meanwhile, this delivery resulted in effient editing at hemoglobin subunit beat loci and rhomboid 5 homolog 1 loci of 19.1% and 7%, respectively [171]. Concerning ocular gene delivery, PEI is one of the most widely investigated polymers. Several studies have explored its potential as an alternative delivery vehicle for the eye [25, 222–225]. For example, one study showed that the intravitreally injected PEI/DNA could be successfully delivered to mouse RGCs [216]. Conceivably, these findings suggest PEI/β-CD as a potential, efficient, and safe nanocarrier for delivering the CRISPR-Cas9 gene editing system into the retina. However, more investigations and efforts are still needed to elucidate the in vivo utility of PEI/β-CD to deliver CRISPR-Cas9 machinery into the retina.

### Inorganic nanoparticles

#### Nanodiamonds (NDs)

**Fig. 4:**
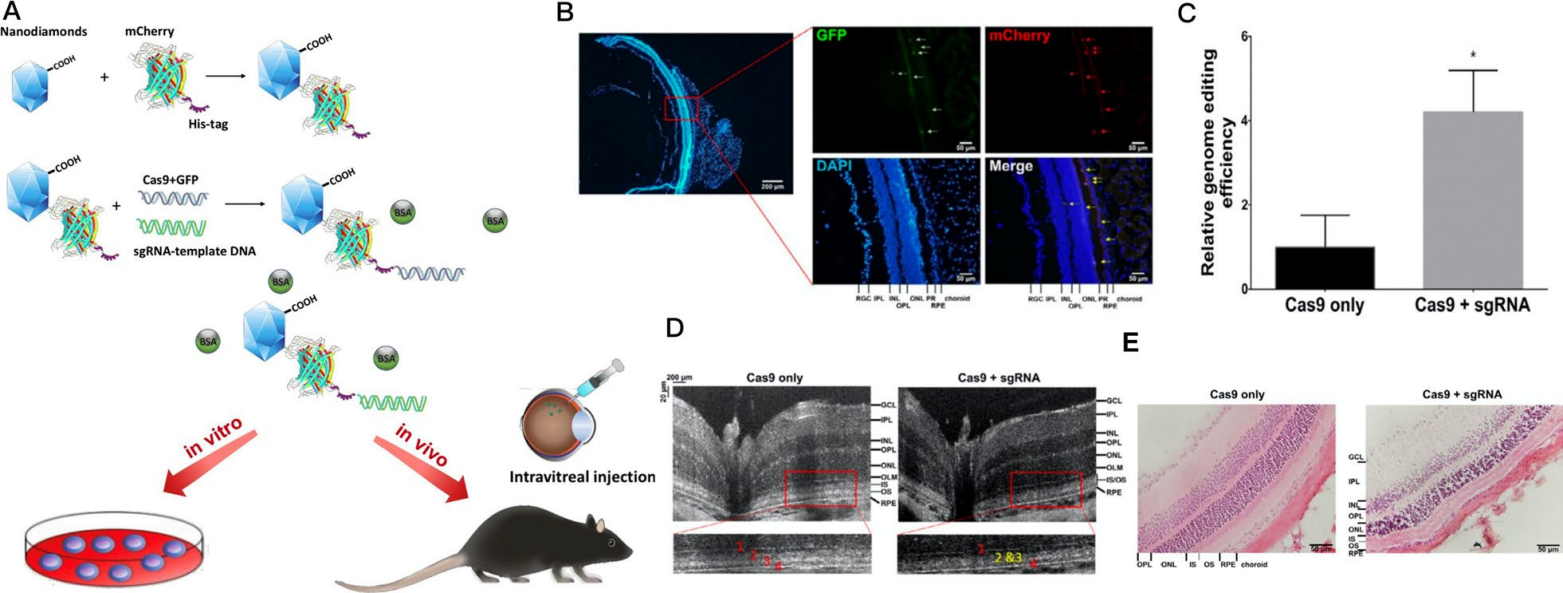
In vivo delivery of the CRISPR-Cas9 machinery using nanodiamond. **A** Functionalized nanodiamond can be covalently bound with mCherry protein and linear plasmid DNA encoding the CRISPR-Cas9 gene editing machinery. After mixing with bovine serum albumin (BSA) at defined conditions, functionalized nanodiamond can promote the delivery of CRISPR-Cas9 machinery in vitro or in vivo to cause precise gene editing. **B** Fluorescence microscopy showing the GFP and mCherry signals in the retinal sections from carboxylated nanodiamond-mCherry- CRISPR-Cas9-treated mouse retina. **C** ddPCR analysis of RS1 c.625 C > T copy number and **D** optical coherence tomography visualization of the mouse retina treated with control (Cas9 only) or carboxylated nanodiamond-mCherry-CRISPR-Cas9 NPs (Cas9 + sgRNA). **E** H&E staining of the retinal sections treated with control (Cas9 only) or carboxylated nanodiamond-mCherry-CRISPR-Cas9 NPs (Cas9 + sgRNA). Visualization methods (**D** and **E**) show the reduction and structural perturbation of photoreceptor inner segment/outer segment layer affected in retinoschisis. Data reproduced from our previous study [239]

Nanodiamonds (NDs) are a novel class of nanomaterials that have garnered a great deal of attention for their clinical application potential due to their low cost, fluorescent capability, low cytotoxicity, and superior biocompatibility [226–231]. It has been reported that most of the NPs destroy the endosomal membrane and release the cargo via the proton sponge effect or chemical reaction [232]. Following the entry of NDs into the cells through endocytosis, the sharp structure of these nanocarriers destroys the endosomal membrane resulting in a quick escape. This unique mechanism of endosomal escape makes NDs more biologically safe and stable [233]. Notably, 2–10 nm NDs provided long-term stability without causing cell death and oxidative stress [231, 234]. In addition, DNA, protein, and drugs can be delivered through different surface modifications of NDs (e.g. carboxylation, hydroxylation, hydrogenation, amination, and halogenation) that improve their intracellular uptake and ability to target specific cells [235–238]. Despite the progress in ND technology, only one study demonstrated the potential therapeutic application of NDs in treating retinal diseases. In our laboratory, we utilized mCherry protein as a critical linker between 3 nm NDs and DNA in which the amide (–NH) and histidine groups (–His) on mCherry protein bind to the carboxylic groups (–COOH) and phosphate groups (–PO_4_^3−^) on the ND and DNA molecules, respectively [239]. These chemical reactions formed a stable link between NDs and the components of the CRISPR-Cas9 genome editing system. The final size of this nanocarrier is about 5 nm, which facilitates its penetration and the delivery of CRISPR-Cas9 components to all layers of the retina, including photoreceptor and retinal pigmentation epithelium layers. The resulting NDs effectively promoted the delivery of the CRISPR-Cas9 components to initiate HDR to direct the c.625C > T mutation of *RS1* gene in human iPSCs and mouse retina, generating an X-linked retinoschisis-like disease model characterized by severe perturbations of the retinal structure (Fig. 4) [239]. The regulatory approval of new drugs by FDA requires inorganic NPs to be cleared via the kidneys to minimize systemic toxicity and improve drug efficacy [240–242]. Since NDs are not biodegradable, using them as the delivery vehicle in therapeutics demands an articulate engineering of NPs to meet FDA standards [231, 243–245]. NDs with the size of 3 nm show a great promise in satisfying the requirements for the drug clearance by the kidneys and the successful transport and release of CRISPR-Cas9 components.

#### Gold nanoparticles (AuNPs)

Gold NPs (AuNPs) are small gold particles with a diameter of 1 to 100 nm that are also known as colloidal gold once suspended in a fluid, usually water [246]. Due to the chemical inertness of gold, AuNPs are not considered as biodegradable materials. However, a previous study conducted more than 3 months of observation and found that AuNPs may be degraded by NADPH oxidase (NOX)-mediated reactive oxygen species (ROS) pathways in human fibroblasts [247]. The biomedical applications of AuNPs have been extensively explored [248–252]. Due to the excellent chemical stability, good biocompatibility, and large specific surface area of AuNPs, they can be a promising alternative for gene delivery, including the CRISPR-Cas9 system. To our knowledge, there are very few in vivo studies that have utilized AuNPs for the delivery of CRISPR-Cas9 system [253–256].

Wang et al. selected AuNPs with a size of approximately 2.4 nm and modified the TAT peptide by glutathione reduction to form the AuNP core. TAT peptide (GRKKRRQRRRPQ) is derived from the human immunodeficiency virus. It is a positively-charged cell-penetrating peptide (CPP) that can overcome the cell membrane's lipophilic barrier and deliver various types of molecules, such as proteins, DNA, antibodies, contrast agents, and toxins. Therefore, AuNP is an attractive approach to deliver the expression plasmids of CRISPR components [257] or the Cas9 protein/gRNA plasmid to target cells [258]. To increase the uptake and delivery efficiency, AuNPs are encapsulated with an anionic lipid shell (1,2-dioleoyl‐3‐trimethylammoniumpropane (DOTAP)/dioleoylphosphatidylethanolamine (DOPE)/cholesterol), which is critical for enhancing the therapeutic application at a low drug dose. The average diameter of the AuNP core is about 20 nm, and the final encapsulated product diameter is about 70 nm (Cas9/gRNA plasmid) or 101 nm (AuNP-CRISPR plasmids).

Although the robust application of AuNP-CRISPR has been found in cancer research, there are some doubts about its application in ophthalmic diseases that need to be further explored. Several in vitro studies reported that AuNP (5–30 nm) may induce cell apoptosis and oxidative stress in the retina [259, 260]. However, in vivo studies did not support the AuNP-induced inflammatory reactions or damage to the retinal structure [261, 262]. Based on the above results, the AuNPs have a potential for gene therapy applications by introducing CRISPR-Cas9 system to the retina. Nonetheless, the excretion, and toxicity of inorganic NPs are important concerns that must be addressed.

#### Graphene oxide (GO)

Graphene, synthesized from graphite, is a nanoscaled monolayer of carbon atoms, arranged in a two-dimensional (2D) crystal structure [263, 264]. Graphene and its family members, including graphene oxide (GO), have been extensively studied owing to their unique properties: high surface area, mechanical and chemical stability, and biocompatibility [264]. Previous studies have found that nano-grade GO, a single-layer graphene oxide sheet, may directly penetrate the cell membrane [265] or enter the cell through endocytosis [266].

Here, we briefly discuss the therapeutic potential of GO nanomaterials in retinal drug delivery. However, only a few studies have utilized GO to deliver CRISPR-Cas9 system [267–271]. Yue et al. constructed a stable and functional dual polymer-GO nanocarrier (GO-PEG-PEI) by conjugating the hydrophilic PEG polymer and the positively charged PEI polymer together with the GO via amide bonds. This dual nanocarrier was utilized to adsorb the negatively charged Cas9/gRNA. The diameter and length of GO-PEG-PEI were 1 nm and 165 nm, respectively, which were increased to 4 nm and 220 nm after loading of the Cas9/gRNA complex. Next, they evaluated the efficiency of GO-PEG-PEI nanocarrier for delivering Cas9/single-guide RNA (sgRNA) complex into the human gastric adenocarcinoma cell line. The results showed 36% of cells underwent NHEJ-based gene editing with 95% cell viability, indicating a successful in vitro gene editing using this nanocarrier [271]. Another in vitro study examined the influence of GO on human retinal pigment epithelium (RPE) cells and demonstrated its satisfactory biocompatibility. However, the cell viability and morphology were slightly affected following a prolnged exposure to GO. Besides, a small amount of GO aggregation was reported [272]. Surprisingly, the intravitreal injection of GO into rabbits’ eyes did not cause obvious ocular structural defects, vision loss, or inflammation [272]. Another study showed that PEG coating strategy promotes the clearance of GO from liver, lung, and spleen in mice, supporting its feasibility for biomedical applications [273]. These results suggested that GO has the potential to carry and deliver CRISPR-Cas9 into the retina; however, the two-dimensional element of GO poses some challenges for its application to treat IRDs.

#### Artificial virus NPs

As mentioned earlier, considerable progress has been made in optimizing CRISPR-Cas9 delivery in vitro. However, further modifications are necessary to improve the delivery efficiency of CRISPR-Cas9 components in vivo. The ligand-receptor interactions strategy is a promising approach for targeted drug and nucleic acid delivery, by which it reduces the drug toxicity and enhances its effectiveness [274]. Since NP’s infection capacity is significantly lower than that of viruses, the strategy of “artificial viruses” was proposed to improve this shortcoming. This strategy takes advantage of a virus-like core (composed of plasmid DNA, condensing agent, and functional peptides) and a hydrophilic shell that can expose specific targeting ligands [275]. Li et al. developed an artificial virus with enhanced endosomal escape and transfection efficiency to successfully carry and release the CRISPR-Cas9 system. In this study, the fluorinated branched PEI (PF33) was utilized as a condensing agent and combined with an expression plasmid containing Cas9 and gRNA directed against the *MTH1* gene as the artificial virus core. The main chain of the shell was natural hyaluronic acid (HA) polymer, and the side chain was modified by PEG and R8-RGD tandem peptide called RGD-R8-PEG-HA (RRPH) multifunctional shell. The combination of PEG side chains with HA backbone improved the artificial virus stability and uptake efficiency. Besides, the terminal attachment of R8-RGD tandem peptide with PEG chain provided the artificial virus with increased target specificity and penetration ability. Notably, the transfection efficiency of the artificial virus was reported as high as > 90% in human ovarian cancer SKOV3 cells, and the resultant CRISPR-Cas9-mediated *MTH1* gene disruption efficiency was 60%. Furthermore, the xenograft analysis of immunodeficient nude mice demonstrated the therapeutic effect of the artificial virus in inhibiting tumor metastasis [276]. So far, there has been no study using this artificial virus nanoparticle in the retina.

#### Supramolecular nanoparticles (SMNPs)

**Fig. 5:**
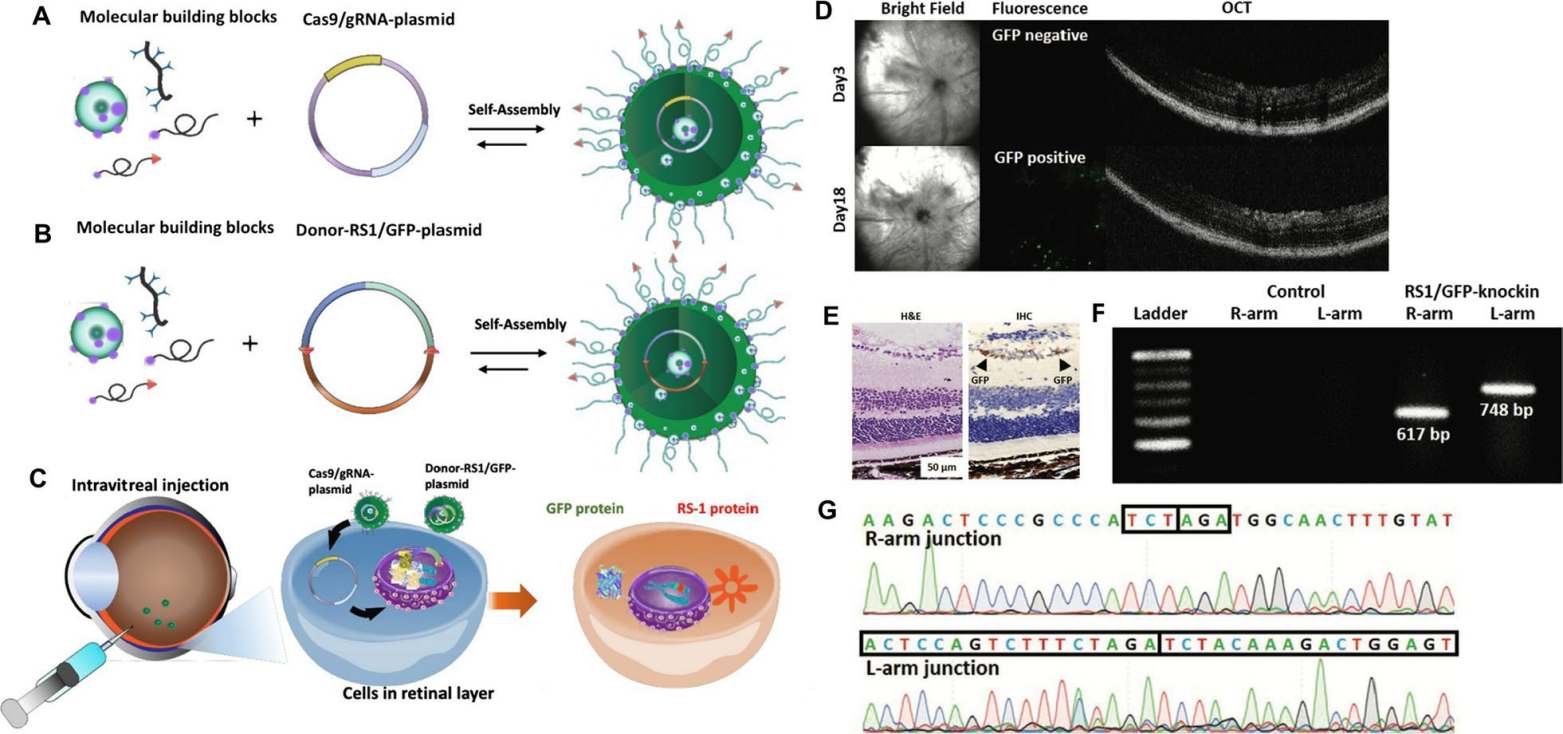
In vivo delivery of the CRISPR-Cas9 machinery using SMNPs. **A**, **B** Schemes of the self-assembly of SMNPs loaded with plasmid DNA encoding Cas9/gRNA or donor-RS1/GFP. **C** A schematic presentation showing the intravitreal injection of SMNPs loaded with the indicated plasmids, resulting in the delivery of CRISPR-Cas9 machinery to express RS1 and GFP proteins in retinal layers. **D** Fundus photography (left) and optical coherence tomography (right) images showing GFP signals and retinal structure after SMNP-mediated gene delivery. **E** H&E and immunohistochemistry staining of GFP-positive cells in the retinal layers. **F** Electrophoretogram showing PCR amplification of the right-arm junction (617 bp) and left-arm junction (748 bp). **G** Sanger sequencing of the genome-donor boundaries showing the effective CRISPR-Cas9-mediated knock-in of RS1/GFP genes in vivo. Data reproduced from our previous study [119]

Another promising nanocarriers for drug delivery with high transfection efficiency are supramolecular nanoparticles (SMNPs). The main features of these NPs are their unique assembly through specific non-covalent interactions and molecular recognition properties that render distinct advantages, including controllable particle size, optimizable surface charge, and enhanced delivery efficiency [277]. The latest SMNP design mainly comprises PAMAM, PEI, and TAT. Among these components, PAMAM produces self-assembled nanoparticles, the cationic polymer PEI condenses DNA, and TAT can penetrate the membrane. In our previous study, we combined PEI/β-CD, adamantane-grafted PAMAM (Ad-PAMAM), adamantine-grafted PEG (Ad-PEG), and Ad-PEG-TAT to form SMNPs to deliver CRISPR components into the retina. To evaluate the efficiency and safety of the designed SMNPs for delivering CRISPR-Cas9 components in gene therapy, they were loaded with donor-RS1/GFP DNA and Cas9/gRNA expression plasmids, resulting in the final size of 110–127 nm. The donor-RS1/GFP was successfully inserted into the ROSA26 (safe harbor) locus using HITI strategy. The delivery efficiencies of donor-RS1/GFP-plasmid using SMNPs and Lipofectamine 3000 were comparable, however, the latter was associated with higher cytotoxicity. This study further showed that SMNPs could deliver the plasmids into the RGC layer via intravitreal injection (Fig. 5A–C) and successfully conducted the knock-in of donor *RS1* into the ROSA26 locus (Fig. 5D–G) [119]. This study introduced a new supramolecular particle with promising features for treating IRDs.

#### Lipid-based cationic nanoparticles (LNPs)

Lipid-based nanoparticles (LNPs) are a mixture of cationic lipids (such as DOTAP, MVL5) and neutral lipids (such as DOPE, cholesterol). These lipids are mixed with DNA under controlled microfluidic conditions and self-assemble into spherical LNPs with a final diameter of 100–200 nm [278, 279]. The cationic lipids are responsible for the electrostatic interaction of LNPs with DNA and their encapsulation [279]. LNPs can be further modified by PEGylation, to reduce the drug aggregation and degradation, which contributes to its therapeutic effect [158]. Additionally, the biodegradability of LNPs can be optimized by modifying the functional groups of ester chains [158].

The transfection efficiency of LNPs is positively correlated with the surface charge density, which is measured by the ratio of cationic lipids to neutral lipids [280]. The high surface charge density of LNPs enables them to effectively fuse with the endosomal membranes, thereby releasing DNA into the cytoplasm [280]. However, the excessive surface charge density hinders DNA dissociation from the liposome complex following endosomal escape [281]. Besides, the excessive positive charge of LNPs causes their poor diffusibility in the negatively charged vitreous as well as their limited passage to the ILM. Although several modifications such as PEG, DOPE, cholesteryl hemisuccinate (CHEMS) have been developed to reduce the positive charge of LNPs [282–284], their safety and transfection efficiency need to be evaluated to expand the utility of gene therapy for IRDs. Despite the challenges mentioned above, LNPs have emerged as a promising alternative for the drug delivery vehicle in clinics [179]. In 2018, Onpattro^®^ (patisiran) was approved by FDA as the first LNP-based nanomedicine used for the treatment of polyneuropathy in patients with hereditary transthyretin-mediated amyloidosis (hATTR amyloidosis) [285, 286]. Later in 2020, LNPs were utilized in FDA-approved COVID-19 vaccines (mRNA-1273 from Moderna, and BNT162b2 from BioNTech/Pfizer) for mRNA delivery [287]. Moreover, D-Lin-MC3-DMA (MC3), an FDA-approved cationic ionizable lipid (CIL)-like LNP, could successfully deliver mCherry mRNA to the RPE layer of the retina in mice [279]. Several in vitro and in vivo studies have demonstrated the application of LNPs for the delivery of the CRISPR-Cas9 system [161–165]. For example, Zhang et al. established a phospholipid-modified cationic lipid nanoparticle (PLNP) delivery system modified with PEG for the delivery of the Cas9/sgRNA plasmid (DNA). Their study demonstrated the delivery efficiency of 47.4% into the A375 cell line (human malignant melanoma), while it was 3.09% for Lipofectamine. Furthermore, the intratumoral injection of PLNP/DNA to a mouse model with a melanoma tumor showed a significant tumor growth reduction [164]. As mentioned earlier, one major concern for the application of CRISPR-Cas9 system in clinics is the uncontrolled expression of Cas9 and gRNA in the cells of interests. Fin et al., created a biodegradable LNP-based delivery system (LNP-INT01) and injected this LNP containing Cas9 and gRNA into mice. The results clearly showed rapid clearance of the molecules 72 h post administration. In addition, they reported a high level (> 97%) knockdown of serum transthyretin levels in mice [161]. These findings are indicative of the efficacy and capacity of LNPs for CRISPR-Cas9 delivery in vivo.

### Other nanoparticles

#### DNA nanoclews

Yarn-like DNA nanoparticles, known as DNA nanoclews, are delivery vehicles synthesized by the rolling circle amplification (RCA) method, producing long-chain ssDNAs with palindromic sequences required for their self-assembly [288]. Since DNA is intrinsically biocompatible and degradable, DNA nanoclews hold great promise in developing ideal drug delivery nanocarriers [289, 290]. However, the immune issues related to these nanocarriers are a matter of concern, therefore, their clinical applications should be further investigated [291]. To the best of our knowledge, no study explored the potential of DNA nanoclews for drug delivery into the eye and only one study reported the utility of DNA nanoclews for delivering CRISPR-Cas9 as briefly described below [292]. Since this type of NPs is made of ssDNA, a sequence complementary to gRNA can be designed to match the base-pair of the guide portion of Cas9-sgRNA [293]. Sun et al. coated the DNA nanoclews/Cas9 protein/gRNA mixture with PEI to improve its cellular uptake and the endosomal escape. The resulting DNA nanoclews were able to deliver the CRISPR-Cas9 components into the target cells in vitro and in vivo with the editing efficiencies of 36% and 25%, respectively [292].

#### Nanoscale zeolitic imidazole frameworks (ZIFs)

Nanoscale zeolitic imidazole frameworks (ZIFs), a subclass of metal–organic frameworks (MOFs), are composed of divalent metal cations and imidazolate bridging ligands with pH buffering capacity. These features enable ZIFs to facilitate endosomal escape [294]. ZIFs combine the advantages of the 3D network and porous structure of zeolite with traditional metal–organic clusters [295, 296] that have recently attracted more attention due to their great potential for delivering drugs, genes, and proteins [297–299]. Alsaiari et al. reported for the first time that ZIF-8 could encapsulate Cas9 protein and gRNA and subsequently undergo genome editing in Chinese hamster ovary (CHO) cells, with loading and editing efficiencies of 17% and 37%, respectively [294]. To the best of our knowledge, no report on the delivery of CRISPR-Cas9 system to the retina using such nanocarrier has been published.

A very recent study utilized a water-in-oil emulsion approach to fabricate a pH-responsive silica–metal–organic framework hybrid NP (SMOF NP) consisting of silica and ZIF. Subretinal injection of SMOF NPs induced efficient genome editing in mouse retinal pigment epithelium. Furthermore, both in vitro loading and delivery efficiencies of CRISPR-Cas9 components by SMOF NPs were high but varied depending on different cell lines [300]. These data introduced a promising nanoplatform that may improve gene therapy in the treatment of IRDs.

To summarize, most modern NPs use non-covalent bonding to carry plasmids expressing Cas9 and gRNA or Cas9 protein and gRNA expression plasmids. These NPs are about 100–200 nm in size with a slight positive charge, which may be suitable for intravitreal injection into the eyes of patients with IRDs. We have also described ND carriers as the only carriers smaller than 10 nm. The size may allow the NPs to pass through the human ILM barrier and effectively perform HDR-based CRISPR gene editing in all retinal layers. Also, SMNPs can adapt to various in vivo environments with their unique molecular recognition ability and therefore have the potential to be an alternative approach for the delivery of CRISPR system to the vitreous. Yet, the means of safe and efficient delivery remain to be fully investigated. The properties and advantages/disatvatages of different types of NPs are summarized in Table 2.

**Table 2:** Summary and comparison of properties of NPs mentioned in this review

Classifications of NPs	NP name	Size (nm)	Materials	Advantages	Disadvantages
Organic NPs	Polymer-based cationic NPs	100–200	Polymers, such as polyethylenimine (PEI), polyamidoamine (PAMAM) dendrimers, chitosan, polyethylene glycol (PEG)	PAMAM enhances the loading capacity, protects the cargo from degradation and lessens systemic toxicity. PEG enhances structural stability, electrostatic binding, and hydrophobicity, and can be tuned to specifically meet the cargo delivery requirements. PEI exploits a large positive surface charge in gene transfection	Some evidence of cytotoxicity and side effects due to residual material aggregation in tissues. Elimination routes and in vivo metabolism have not been elucidated
Inorganic NPs	Nanodiamonds (NDs)	2–8	Carbon with truncated octahedral architecture	Low cost, fluorescent capability, low cytotoxicity, and provides long-term stability without causing cell death and oxidative stress	There exist differences and complications in characterizing NDs in dry state and in living organisms. Few in vivo studies done
Inorganic NPs	Gold NPs	1–100	Gold	Excellent chemical stability, good biocompatibility, tunable size, and large specific surface area	More information about uptake, biocompatibility and low cytotoxicity is required for clinical translation
Inorganic NPs	Graphene oxide NPs	165	A single-layer graphene oxide sheet (graphene is a layer of carbon arranged in a 2D crystal structure)	High surface area, mechanical and chemical stability, and biocompatibility. Able to directly penetrate the cell membrane or enter the cell through endocytosis	Some evidence of dose-dependent cytotoxicity and cell apoptosis
Inorganic NPs	Artificial virus NPs	130–135	Virus-like core (composed of plasmid DNA, condensing agent, and functional peptides) and a hydrophilic shell	Improved infection capacity; naturally occurring nanomaterials and hence biocompatible and biodegradable	Further modifications are necessary to improve the delivery efficiency in vivo
Inorganic NPs	Supramolecular NPs (SMNPs)	110–127	PAMAM, PEI, and TAT, assembled through specific non-covalent interactions and molecular recognition properties	Tunable particle size, optimizable surface charge, and enhanced delivery efficiency	More information about uptake, biocompatibility and low cytotoxicity is required for clinical translation
Inorganic NPs	Lipid-based cationic NPs	100–200	A mixture of cationic lipids (such as DOTAP, MVL5) and neutral lipids (such as DOPE, cholesterol)	Low toxicity, biodegradable, able to transport both hydrophobic and hydrophilic molecules	Crystallize after prolonged storage conditions, and poor diffusibility in the negatively charged vitreous due to excessive positive charges
Other NPs	DNA Nanoclews	56	Long-chain ssDNAs with palindromic sequences	Intrinsically biocompatible and degradable	More information about immune- related issues is required for clinical translation
Other NPs	Nanoscale zeolitic imidazole frameworks (ZIFs)	100–200	Made of divalent metal cations and imidazolate bridging ligands	3D network with a porous structure that facilitates endosomal escape	No known report on this delivery system for the retina yet

## The retinal organoids and precision medicine

The heterogeneity of IRDs hampers the development of an effective strategy to tackle a wide range of disorders. One of the major hurdles that hinders the translation of basic retinal research into clinical applications is mainly due to the poor relevance in existing preclinical models. For example, in the mouse model, more than 90% of photoreceptors are rod cells, whereas, in humans, the visual acuity is mostly dependent on cone photoreceptors [301]. Notably, many in vitro and in vivo findings could not be reproduced in humans. Drugs that proved to be safe and effective in animal studies failed to exert the same efficacy in clinical trials. In addition, the information obtained by studying two-dimensional cultures does not recapitulate the heterogeneous complexity and critical features of the microenvironment of cells in vivo [136–138]. This gap causes a noticeable lack of fidelity between the aforementioned experimental models and human outcomes. The advent of three-dimensional (3D) multicellular constructs, referred to as the organoid technology, offers a promising complementary model to pursue clinical translation and precision medicine applications. Human pluripotent stem cells (hPSC)-derived retinal organoids hold an excellent value for modeling the human retina features [302–306]. In particular, by using patient-derived cells combined with reprogramming strategies, this technique could represent an efficient pre-clinical approach toward the personalized therapeutic strategies adaptable to a broad number of IRDs and provide the link to disease-specific human drug screening models [307]. The iPSC-derived organoids strategy can provide a means for assessing the efficiency and efficacy of NP-mediated delivery of CRISPR-Cas9 system tailored to each patient's genetic makeup. In this part, we mainly focus on organoid as a technology platform for precision medicine in IRDs, especially with potential translational applications for evaluating new therapeutic drugs.

### Organoids as a drug testing platform for translational research

Introducing a new pharmaceutical drug to the market is a complicated and costly process, especially when the in vivo testing result of drug candidates fail to reach the requirements initially fulfilled by the in vitro test. The gap between in vitro validation and clinical application is significant, mainly because the simplicity of the in vitro model cannot mimic the complex nature and heterogeneous characteristics of clinical patients [308]. These critical problems impose significant limitations on the translation of candidate drugs to the clinic and require advanced strategies to improve this shortage. Organoid systems show considerable reliability of recapitulating features and functions of the human system offering a great potential for testing drug efficiency in target organs. Patient-derived organoids (PDOs) can be generated particularly by reprogramming patient-derived cells to induced pluripotent stem cells (iPSCs), followed by the differentiation into the desired cell lineage and organoids [309]. Notably, several reports showed that in vitro PDOs could highly match and reproduce patients’ response to candidate drugs in most cases, highlighting the merit of this system in personalized medicine as a predictor of therapeutic outcome [310–313]. The organ-like structure technology offers a more efficient screening of candidate drugs prior to in vivo testing, which helps to reduce drug development costs. Organoids have been a powerful tool for functional drug testing, personalized therapy and disease modeling [314–320]. For iPSC-derived organoids, various organoid models have been generated using human iPSCs, including heart [321, 322], kidney [323], brain [324, 325], intestine [312, 313, 326], liver [327–329], lung [330] and retina [302–305]. Furthermore, organoids have been successfully applied to model human genetic diseases. For example, intestinal organoids derived from cystic fibrosis (CF) patients proved to be a reliable tool for effective drug treatment [314]. Of note, brain [315–317] and kidney [318–320] organoids generated from patient-derived iPSCs have also been established to model diseases. Xu et al. subjected brain organoids to Zika virus infection and used them as the platform for drug repurposing [331]. Bian et al. demonstrated that the neoplastic cerebral organoids are suitable for targeted drug testing [332]. Saengwimol et al. used retinoblastoma organoids for the evaluation of cellular response to chemotherapy drugs. [333]. However, the consistency and reducibility of this system at a scale consistent with clinically associated cell numbers is still a matter of concern [334]. For retinal organoids, Vergara et al. developed an iPSC-derived retinal organoid-based screening platform that allows the accurate quantification of fluorescent reporters [335]. Despite the progress of some organoid-based researches, the organoid technologies remains immature and not ready for the demands of high-throughput screening in drug screening [336]. It was attributed to the developmental variability and diversity of retinal organoids that may hinder the utility of retinal organoids in the evaluation of therapeutic effects and comparative analysis [337]. Nevertheless, considering that retinal organoids hold promising potential in new drug development, it would be still expected and encouraging to use retinal organoid technologies to augment the existing drug development pipelines. Collectively, these findings highlight the utility of organoids as a part of the drug testing pipeline, creating the opportunities for more effective therapies, especially for patients with rare genetic diseases in a cost-effective manner.

### Retinal organoid applications in precision medicine

**Fig. 6:**
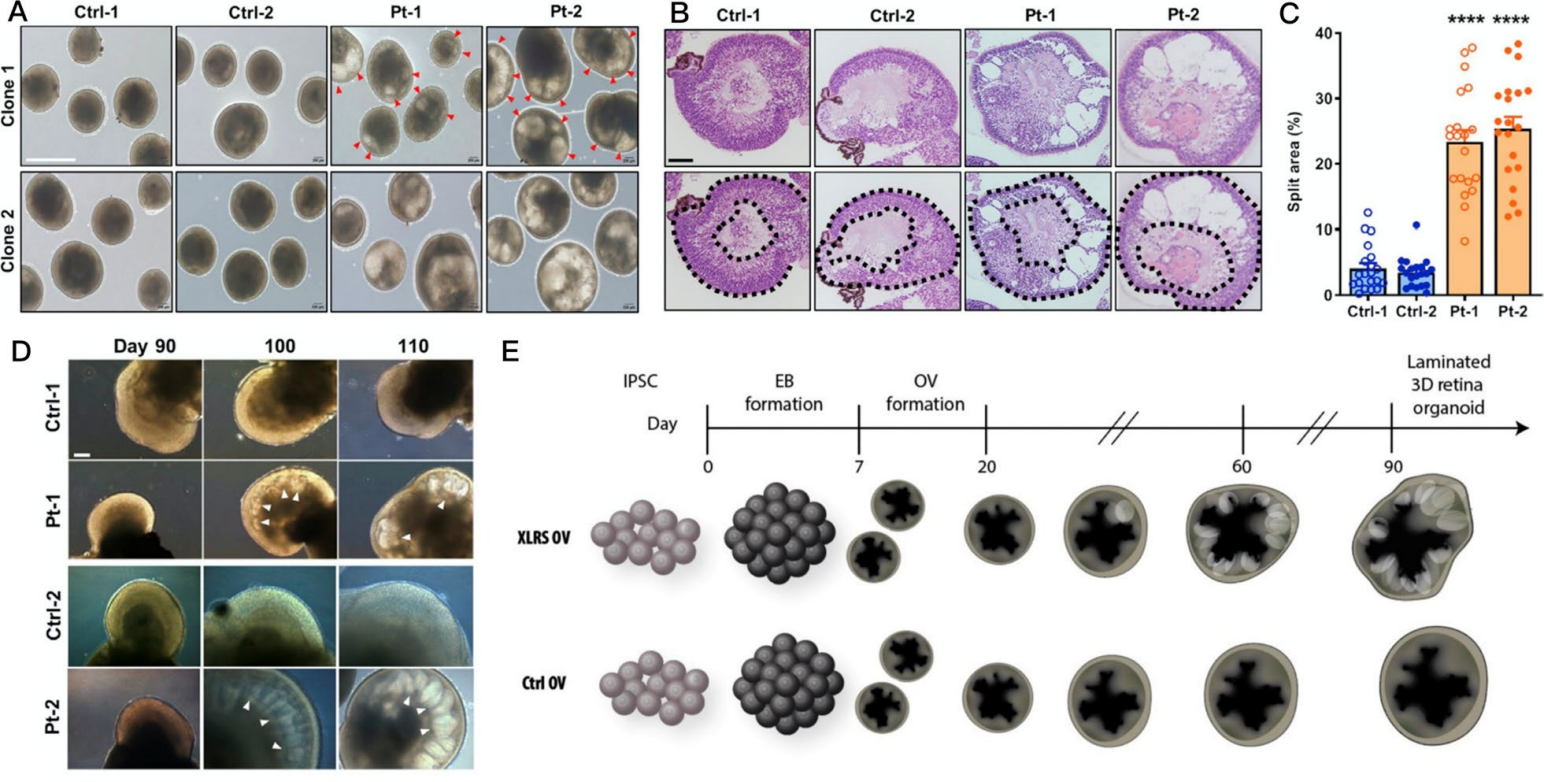
Patient-derived retinal organoids recapitulate disease-specific features. **A** Bright-field images and **B** H&E staining of control and XLRS patient-derived retinal organoids exhibit schisis feature at day 150 of differentiation. **C** Quantification of splitting area in control and XLRS-patient-derived retinal organoids. **D** Bright-field images of control and XLRS patient-derived retinal organoids at days 90, 100, and 110 of differentiation. **E** A schematic presentation of the time course for the generation of control and XLRS patient-derived retinal organoids. Disease-specific features can be observed after applying defined differentiation stimuli and time course. Reproduced from our previous study [30]

To establish a drug testing platform for IRD treatment based on organoid technologies, generating a representative disease model is a fundamental step. As highlighted below, several reports demonstrated the utility of organoids in eye disease modeling. Ohlemacher et al. developed a retinal organoid model using patient-specific iPSC-derived RGCs to study an inherited form of glaucoma [26]. Tucker et al. generated multi-layer optic cup-like structures representing photoreceptor precursor cells for investigating the pathogenesis of RP [27]. A separate study focused on different frameshift mutations in the *RPGR* gene, one of the most prevalent causes of autosomal recessive RP, and generated patient-specific retinal organoids with defects in morphology and functionality of photoreceptors accompanied with decreased cilia length as a disease model [28]. The constructed vectors for the CRISPR-Cas9 machinery were delivered into the patient-derived iPSCs via electroporation, and the mutation-corrected iPSCs were then differentiated into retinal organoids. Notably, the reversal of morphological and functional defects in retinal organoids with *RPGR* mutation was observed after the CRISPR-Cas9-mediated gene correction [28]. Using a similar approach, Buskin et al. demonstrated the severe RP defects observed in patient-specific retinal organoids harboring the CRISPR-Cas9-induced *PRPF31* mutation [29]. This further proved the effectiveness of this combined strategy toward personalized and targeted gene therapy. Another example for coupling retinal organoids with the genome engineering technique is the application of CRISPR-Cas9 technology to correct *RS1* mutation in retinal organoids derived from XLRS-patients [30]. Huang et al. successfully established the XLRS patient-derived retinal organoids that recapitulate the retinal splitting feature of the disease. Meanwhile, they delivered the CRISPR-Cas9 system using electroporation to correct the mutation and showed that CRISPR-Cas9-mediated correction of the disease-associated C625T mutation efficiently rescued the disease phenotype (Fig. 6) [30]. Parfitt et al. used LCA patient-derived iPSCs to generate 3D optic cups with the mutation of a cilia-related gene, *CEP290* [31]. Introducing an antisense oligonucleotide to patient iPSC-derived organoids could effectively prevent the aberrant splicing, restore the expression of full-length CEP290 protein, and repaired the cilia defects [31]. Currently, *CEP290* treatment is in phase III clinical trial (NCT03913143), paralleling the classic augmentation *RPE65* trials initiated in 2007 [338, 339]. Overall, these studies demonstrated that 3D retinal organoids derived from patients with various retinal diseases are able to recapitulate the complex retinal architecture, rendering them an ideal platform for examining the safety and specificity of CRISPR-Cas9 system for the therapeutics applications.

## Conclusions and perspectives

### The combination of NPs, CRISPR-Cas9, and retinal organoids as a promising therapeutic platform

**Fig. 7:**
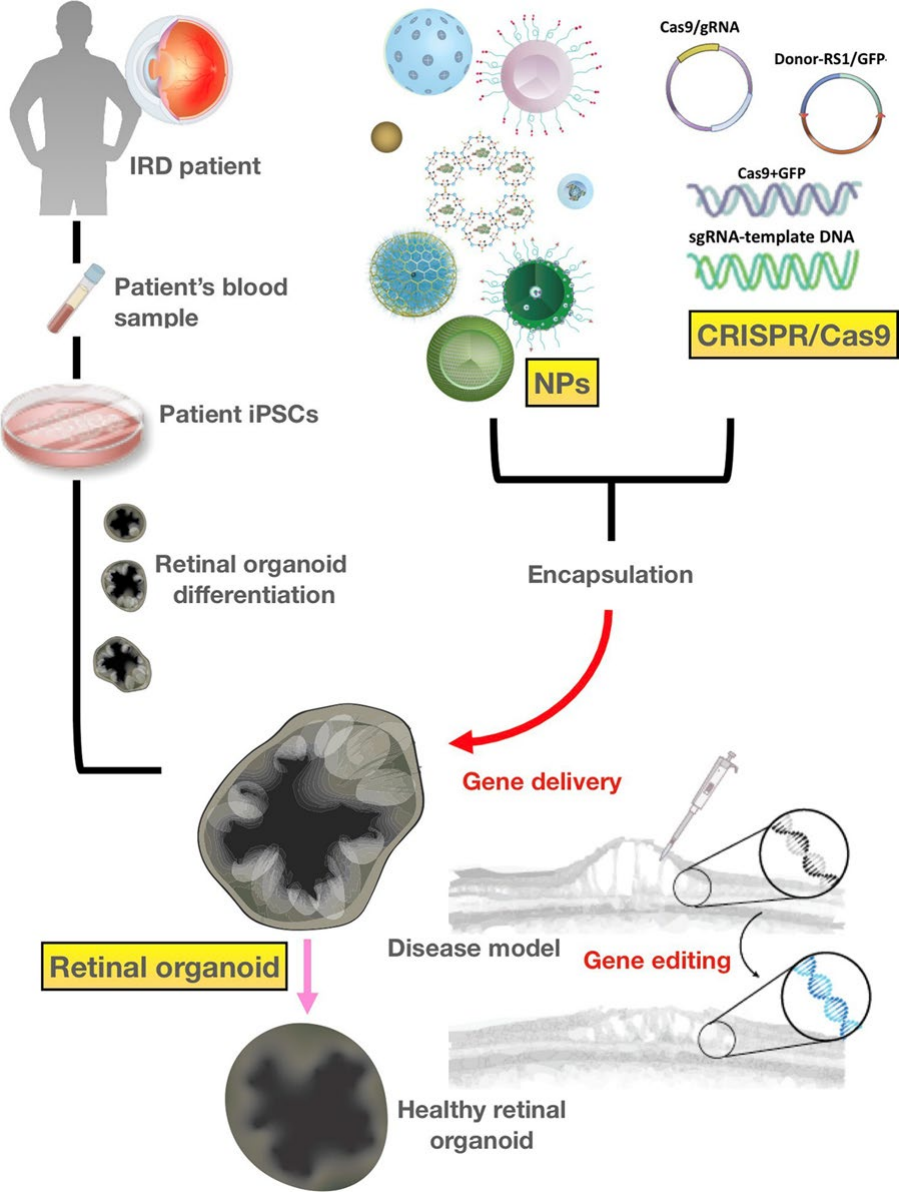
An integrative, multidisciplinary approach for future gene therapy in IRDs. IRD patient’s blood sample can be reprogrammed into induced pluripotent stem cells (iPSCs) followed by the differentiation into retinal organoids. These patient-derived retinal organoids exhibit disease-specific features and can be applied as a reliable platform for assessing disease progression and treatment outcome (e.g. XLRS-patient-derived retinal organoids exhibit severe retinoschisis-like features). Researchers can utilize optimized NPs loaded with plasmid DNA encoding CRISPR-Cas9 machinery to achieve efficient gene delivery and precise gene editing. This results in the rescue of the disease phenotypes associated with the specific IRDs (e.g. the splitting phenotype in XLRS-patient-derived retinal organoids can be rescued as shown above). Integrating patient-derived retinal organoids, CRISPR-Cas9 technologies, and NPs promotes precision gene therapy applications for IRDs

IRDs have long been viewed as a class of disorders with no effective treatment. This maxim is now being reversed by tremendous efforts in nanomedicine and gene engineering, which results in promising clinical trials for blinding diseases. As therapeutic strategies for IRDs expand, the importance of molecular diagnosis is gaining momentum. Most IRD gene supplementation therapies are in phase I/II clinical trials, with LCA therapy approved by the FDA to treat patients carrying biallelic *RPE65* mutations [59, 62, 340–342]. Although these efforts are still evolving, the importance of gene therapy for elevating the life quality of IRD patients has never been more apparent. In this review, we introduced a forward movement of therapy by combining the advances in CRISPR-mediated gene editing, NP-based delivery, and iPSC-derived retinal organoids technologies, to assess the potential safety and efficacy of designed CRISPR-Cas9 components and nanocarriers in a clinically relevant in vitro model. As mentioned earlier, NP-mediated delivery of high molecular weight CRISPR-Cas9 complexes combined with the advanced CRISPR-Cas9 technologies, applicable in non-dividing retinal cells (e.g. HITI), introduces a simple yet efficient approach for precise gene therapy in IRDs. In addition, patient-derived retinal organoids can mimic typical disease features, providing a reliable platform for disease modeling. Collectively, this integrated strategy is expected to facilitate the evaluation of the gene editing in preclinical tests and be a major driver towards advancing IRD’s personalized medicine (Fig. 7).

During the past few years, our preliminary research on combining CRISPR-Cas9 gene editing, patient-derived retinal organoids, and NPs has been conducted to investigate the potential of this integrated strategy in IRD therapeutics. For example, Yang et al. utilized ND-mediated delivery of CRISPR-Cas9 to introduce the mutated *RS1* gene into human iPSCs and mouse retina, leading to the generation of XLRS-like disease model [239], however, the potential of this strategy for gene therapy is yet to be explored. In another study, Chou et al. successfully combined SMNP nanocarrier and CRISPR-Cas9-mediated HITI strategy to knock-in the *RS1* gene in the mouse retina [119]. In the future, this strategy can be further applied in patient-derived retinal organoids model to assess its efficiency and efficacy in clinically relevant disease setting. In a separate study, Huang et al. successfully differentiated iPSCs from XLRS patients into retinal organoids presenting disease features. They further coupled this disease model with CRISPR-Cas9 technology and repaired the *RS1* gene mutation with 50% efficiency [30]. However, the electroporation method used for the transfection of CRISPR-Cas9 machinery caused a massive cell death [30]. Therefore, future attempts to utilize NPs as an alternative method will be of great interest to resolve this drawback and further boost the therapeutic effects. We hope that this combined strategy will become a treatment modality for other IRDs and elevate the life quality of patients.

### Technology hurdles

Although the rationale for this integrated approach is clear, several technology hurdles remain to be addressed. The development of advanced CRISPR-Cas9 systems with high specificity has armed researchers worldwide with a powerful tool to study human diseases. However, utilizing this technique for translational medicine research has inevitable concerns. Since patient-derived iPSCs not only carry the specific mutation intended to be repaired but also harbor the entire human genome, this makes CRISPR-Cas9-mediated gene editing more susceptible to undesired off-target effects. For example, gRNA may recognize sequences similar to the target loci and cause permanent sequence alterations resulting in abnormal gene function. Furthermore, no in vivo study examined how long the nuclease remains active before its degradation and what the possible adverse effect(s) might be. Anti-CRISPR proteins can be used to limit the off-target editing, however, the best time to shut off Cas activity requires optimizations [343]. Another concern is that CRISPR-Cas9-mediated knockout or overexpression of the gene of interest may be compensated by neighboring cells, which would interfere with the expected outcome [344]. As for the organoid technology platform, although the 3D retina organoid has equipped ophthalmology with a unique and relatively accurate representation of the human eye, it still lacks a high level of morphological and functional complexity demonstrated by the mammalian retina in vivo. For example, the 3D retinal organoid with retinal pigment epithelium (RPE) layer provides a more physiologically relevant disease model for photoreceptor-associated diseases. However, both simple and complex organoid models have their pros and cons; thus, the appropriate level of complexity should be designed according to the purpose of the study [345]. A more challenging issue is the disease modeling of late-onset retinal diseases by manipulating culture medium to induce ageing factors. It demands a profound knowledge of factors involved in each specific developmental stage which are yet to be investigated. More in-depth knowledge and assessment of the culture medium composition and distribution are required for modeling complex IRDs. Nevertheless, retinal organoid technologies have only been utilized for monogenic IRDs so far. Lastly, although NP-based delivery has been proposed and proven to be a promising drug delivery vehicle, improving the therapeutic application of NP-based gene therapy remains an important concern. It requires in-depth investigation on the cytotoxicity of the NPs under variable conditions and on the key factors determining the release rate of drugs from NPs to the retina. The successful drug delivery to the neuroretina, and even more specifically to photoreceptors, highly depends on the choice of the NPs most suitable for both the drug and the target tissue. Besides, different gene mutations may affect the complex retina structure and cause anatomical obstacles for nanomedicine drug delivery. Moreover, the choice of delivery route, immunoreactivity, and nucleic acid-based drug stability are critical factors that need to be addressed for a successful clinical application. Nevertheless, in this ever-evolving field, it is crucial to move scientific discoveries into clinics and new therapies for vision restoration in more patients than ever before. Ultimately, the application of such technologies in the clinic and industry should fulfill four criteria: reproducibility, standardization, validation, and quality assurance. Although NP-mediated delivery of the CRISPR-Cas9 system shows a great promise in repairing the IRDs, the time window for treatment is a critical determinant for the therapeutic outcome. For example, in XLRS, the differentiated retinal cells with the splitting phenotype are not responsive to gene therapy, indicating the delayed treatment by the time the disease is already progressed. However, more preclinical data will be required to prove this concept. Taken together, the advances and current progress of basic research hold the promises that laboratory findings can be translated into clinical applications in the near future and bring hope to patients who have blindness and other hereditary diseases. The integration of nanotechnology, CRISPR, and stem cell technologies present a novel platform and is expected to accelerate bridging the basic research and translational medicine, and further promote medical precision therapies.
